# Correction: Longitudinal dynamics of SARS-CoV-2-specific cellular and humoral immunity after natural infection or BNT162b2 vaccination

**DOI:** 10.1371/journal.ppat.1013976

**Published:** 2026-02-19

**Authors:** The PLOS Pathogens Editors

**Notice of Republication**

This article [1] was republished on January 16, 2026, to address an issue identified post-publication. An updated version of S1 Raw Data is provided with this notice. Please download this article again to view the correct version.

## Supporting information

S1 Raw Data Database including all numerical values that were used to generate graphs and statistics. (XLSX)
