# Supplementary figures and images for: Longitudinal dynamics of SARS-CoV-2-specific cellular and humoral immunity after natural infection or BNT162b2 vaccination
Source: PLoS Pathog. 2021 Dec 28;17(12):e1010211. doi: 10.1371/journal.ppat.1010211 (PMC8757952; doi:10.1371/journal.ppat.1010211)

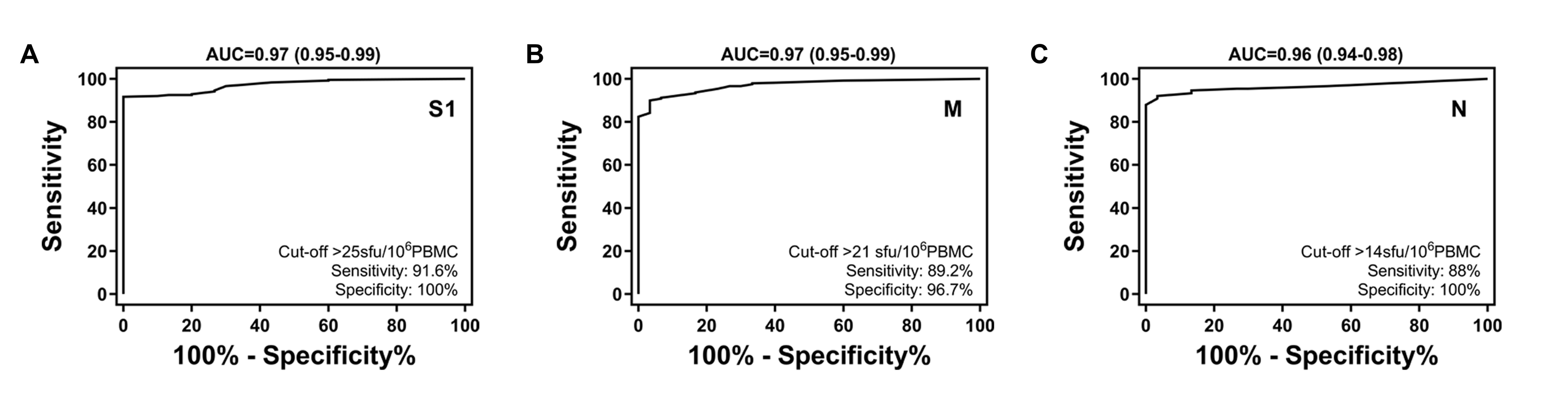

Supplement: S1 Fig — Cut-off values were established by using a control group of 30 healthcare workers with no microbiological or clinical evidence of SARS-CoV-2 infection and 234 recovered COVID-19 patients. (A) S1 Cut-off >25 IFN-γ sfu/106 PBMC (AUC = 0.97, 95%CI = 0.95–0.99). (B) M Cut-off >21 IFN-γ sfu/106 PBMC (AUC = 0.97, 95%CI = 0.95–0.99). (C) N Cut-off >14 IFN-γ sfu/106 PBMC (AUC = 0.96, 95%CI = 0.94–0.98). AUC: Area Under the Curve; IC: Confidence Interval. (TIF) [file ppat.1010211.s001.tif]

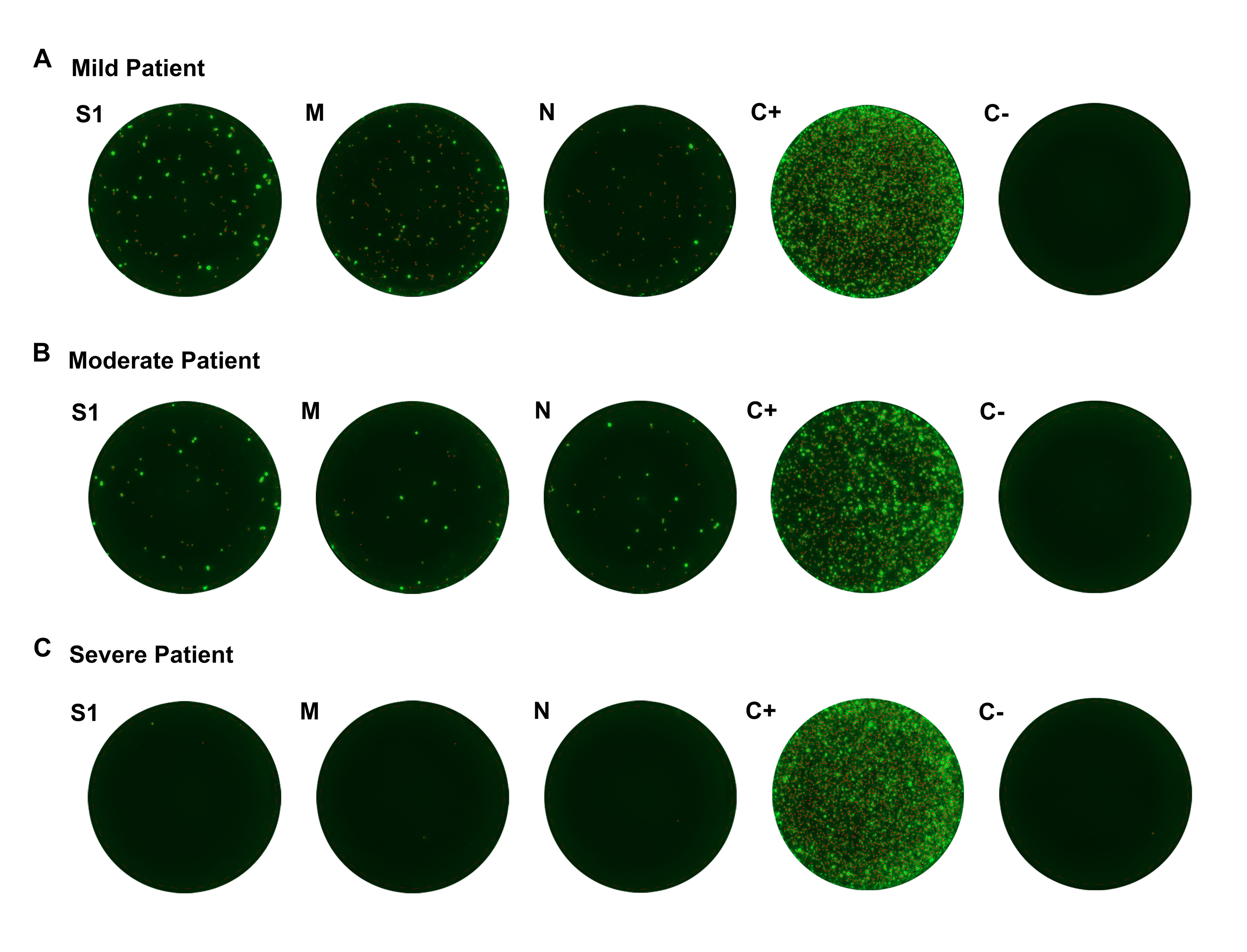

Supplement: S2 Fig — FluoroSpot IFN-γ responses against SARS-CoV-2 S1, M and N peptide pools and positive (anti-CD3 and anti-CD28 mAb) and negative (anti-CD28 mAb only) control wells for mild (A), moderate (B) and severe (C) patients. (TIF) [file ppat.1010211.s002.tif]

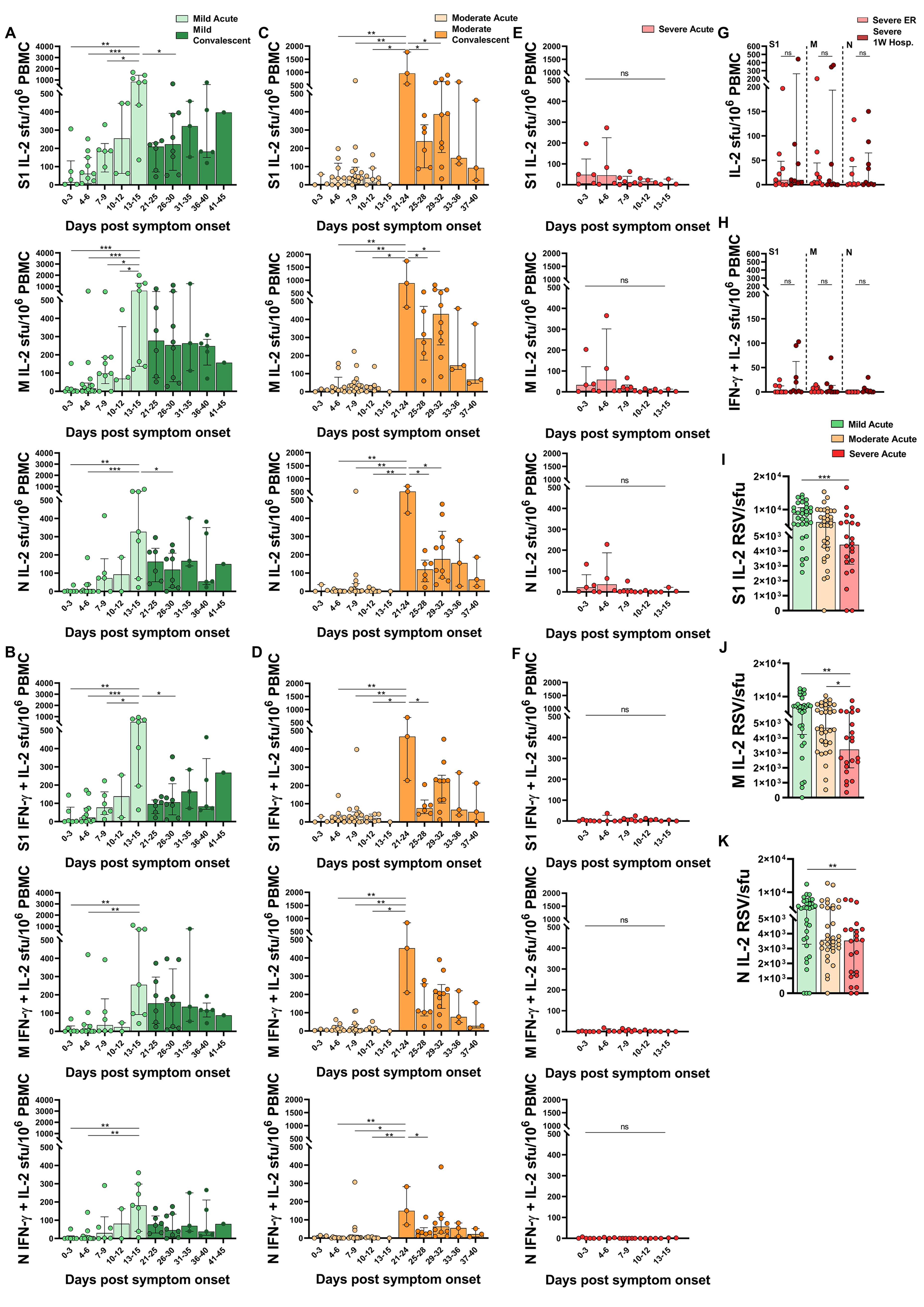

Supplement: S3 Fig — IL-2 and bifunctional IFN-γ+IL-2 T cell responses against SARS-CoV-2 S1, M and N protein pools according to the days post symptom onset during acute and convalescent phase in mild (green) (A-B) moderate (orange) (C-D) and severe (red) (E-H) patients. Data is represented as spot forming unit (sfu) per million PBMC. (I-K) Relative Spot Volume (RSV) per spot of secreted IL-2 after S1, M and N peptide pool stimulation in mild, moderate, and severe patients during acute infection. Horizontal bars and whiskers represent median values and interquartile ranges, respectively. The significance between groups was determined using Mann Whitney, Wilcoxon signed rank or Kruskal-Wallis tests, *p<0.05, **p<0.01, ***p<0.001, ****p<0.0001. (TIF) [file ppat.1010211.s003.tif]

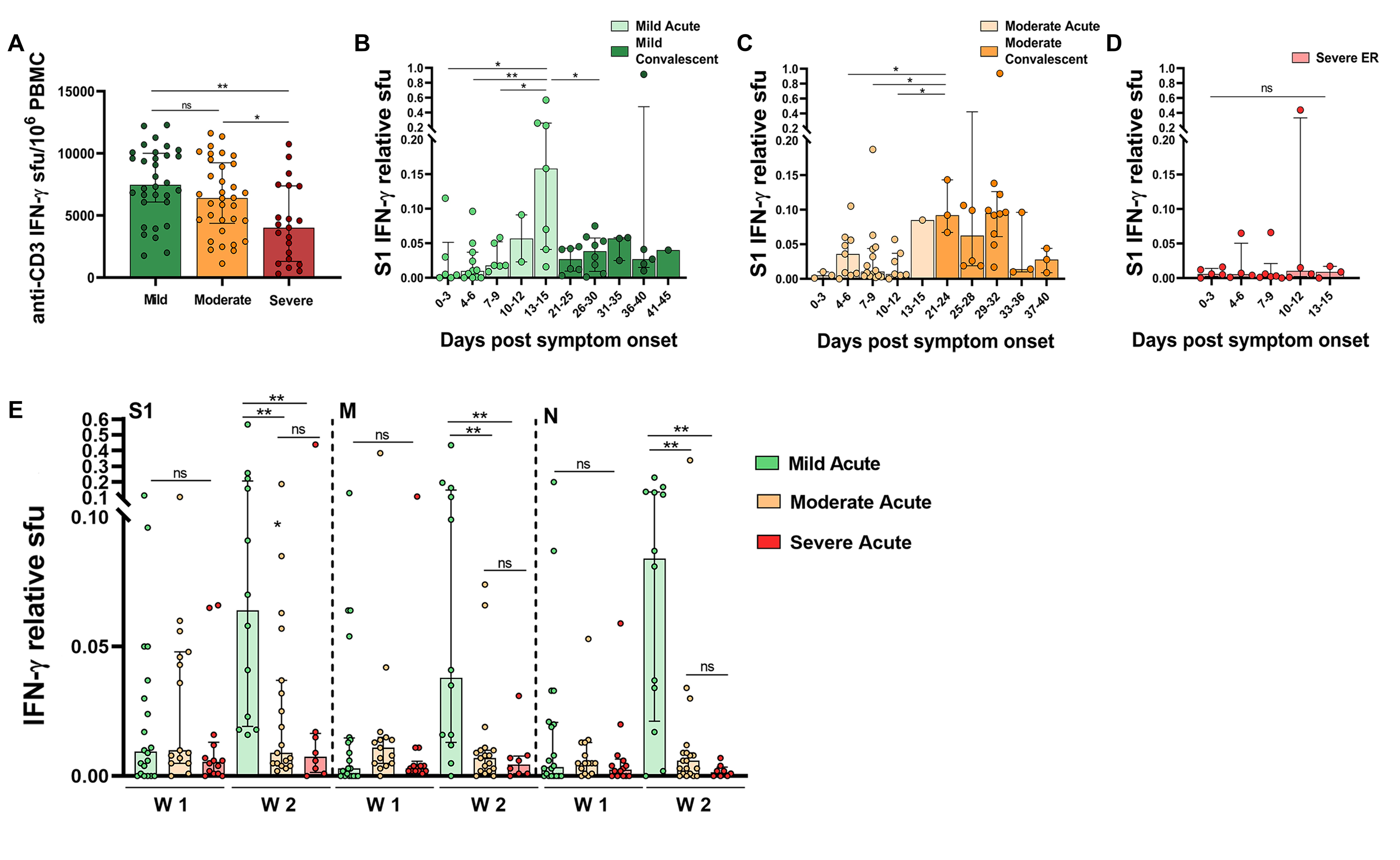

Supplement: S4 Fig — (A) FluoroSpot IFN-γ responses against anti-CD3mAb polyclonal stimulus in mild (green), moderate (orange) and severe (red) COVID-19 patients. Data is represented as spot forming unit (sfu) per million PBMC. (B-D) S1, M and N cellular response adjusted by anti-CD3 sfu/106PBMC normalization according to the days post symptom onset during acute and convalescent phase in mild (green), moderate (orange) and severe (red) COVID-19 patients. Data is shown as relative sfu per million PBMC. (E) Relative SARS-CoV-2-specific IFN-γ-producing T-cell responses reactive to the S1, M and N proteins in mild, moderate, and severe patients during the first (W1) and the second (W2) week post symptom onset. Horizontal bars and whiskers represent median values and interquartile ranges, respectively. The significance between groups was determined using Mann Whitney, Wilcoxon signed rank or Kruskal-Wallis tests, *p<0.05, **p<0.01, ***p<0.001, ****p<0.0001. (TIF) [file ppat.1010211.s004.tif]

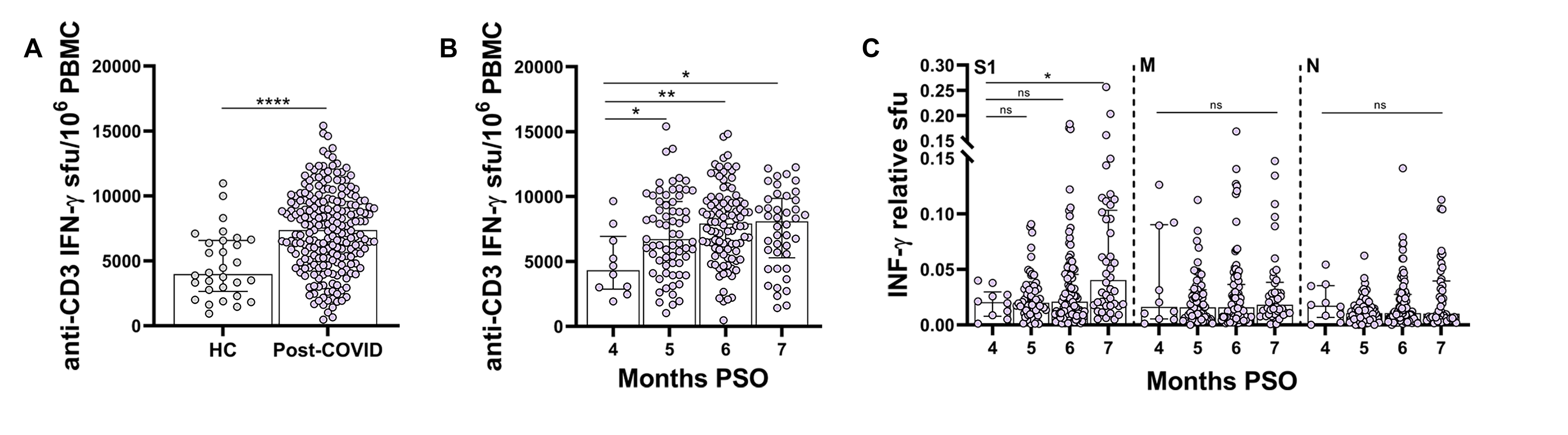

Supplement: S5 Fig — (A) Comparison of FluoroSpot IFN-γ responses against anti-CD3mAb polyclonal stimulus in healthy controls (HC) and moderate or severe recovered patients (Post-COVID). (B) anti-CD3mAb IFN-γ responses according to the months post symptom onset (PSO). (C) S1, M and N cellular response normalized by anti-CD3 sfu/106PBMC, according to the months PSO. Data is shown as relative sfu per million PBMC. Horizontal bars and whiskers represent median values and interquartile ranges, respectively. The significance between groups was determined using Mann Whitney test, *p<0.05, **p<0.01, ***p<0.001, ****p<0.0001. (TIF) [file ppat.1010211.s005.tif]

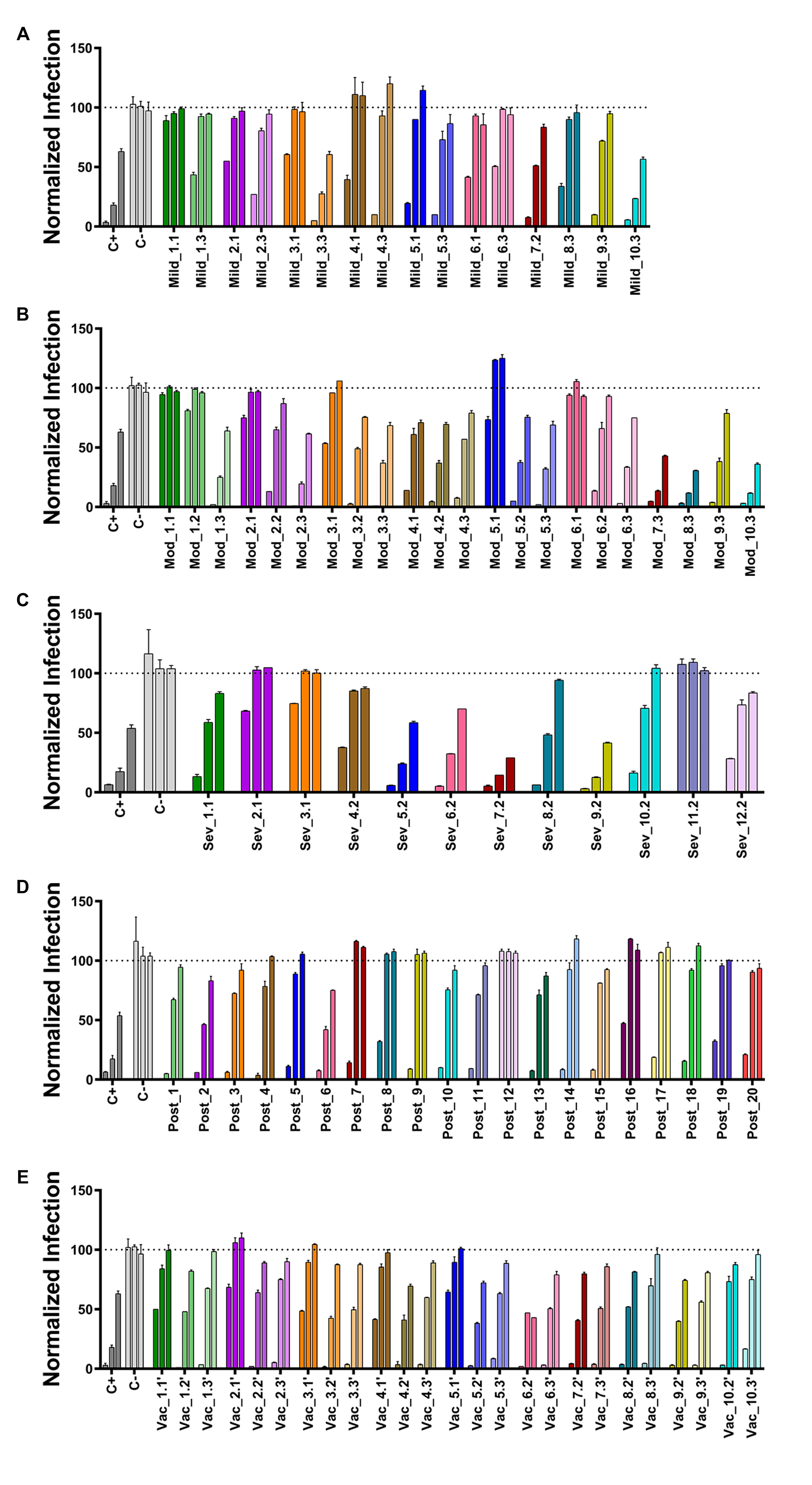

Supplement: S6 Fig — Neutralization capacity represented as normalized infection achieved in the presence of 1/50, 1/500 and 1/2000 (from left to right) serum dilutions for each sample in (A) Mild COVID-19 patients; (B) Moderate COVID-19 patients (Mod); (C) Severe COVID-19 patients (Sev); (D) Moderate or severe COVID-19 recovered patients (Post) and (E) after BNT162b2 vaccination (Vac). Coding sample in natural infection, 1: acute phase; 2: hospitalization; 3: convalescent phase. Coding sample in vaccination, 1’: pre-boost; 2’: 15 days after complete vaccination; 3’: 30 days after complete vaccination. Horizontal bars and whiskers represent median values and interquartile ranges, respectively. (TIF) [file ppat.1010211.s006.tif]
