# Supplementary material for: Longitudinal dynamics of SARS-CoV-2-specific cellular and humoral immunity after natural infection or BNT162b2 vaccination
Source: PLoS Pathog. 2021 Dec 28;17(12):e1010211. doi: 10.1371/journal.ppat.1010211 (PMC8757952; doi:10.1371/journal.ppat.1010211)
Supplement: S1 Table — (DOCX) [file ppat.1010211.s007.docx]

**SUPPLEMENTARY TABLES**

**S1 Table. Lymphocyte populations in successive phases of the disease according to severity**

| Disease course/severity | | Acute phase | | | | | |
| --- | --- | --- | --- | --- | --- | --- | --- |
|  |  | **CD3** | **CD4** | **CD8** | **CD4/CD8** | **B** | **NK** |
| Mild | Median cell/µl (IQR) | 1533 (1292-1820) | 662 (575-894) | 386 (259-443) | 1.72 | 157 (110-232) | 197 (153-250) |
|  | % (IQR) | 75% (71-80%) | 47% (43-50%) | 24% (20-27%) |  | 10.3% (9-13%) | 13% (9-16%) |
| Moderate | Median cell/µl (IQR) | 817 (645-1154) | 328 (220-437) | 185 (130-266) | 1.78 | 99 (67-113) | 161 (78-296) |
|  | % (IQR) | 67% (60-72%) | 37% (30-48%) | 23% (17-29%) |  | 11% (8-15%) | 18% (12-27%) |
| Severe | Median cell/µl (IQR) | 437 (103-629) | 251 (68-341) | 177 (35-204%) | 1.42 | 102 (22-133) | 80 (48-140) |
|  | % (IQR) | 59% (51-70%) | 39% (29-40%) | 21% (20-23%) |  | 12.8% (11-16%) | 15.4% (11-20%) |
| Count (cell/µl) p-value | | <0.0001 | <0.0001 | 0.0002 |  | 0.003 | 0.02 |
| Disease course/severity | | **Convalescent phase** | | | | | |
|  |  | **CD3** | **CD4** | **CD8** | **CD4/CD8** | **B** | **NK** |
| Mild | Median cell/µl (IQR) | 2001 (1790-2816) | 864 (1006-2046) | 499 (419-710) | 1.73 | 246 (162-361) | 263 (197-342) |
|  | % (IQR) | 76% (69-78%) | 45% (41-51%) | 25% (22-29%) |  | 12% (10-15%) | 13% (10-15%) |
| Moderate | Median cell/µl (IQR) | 1553 (1153-1921) | 621 (480-756) | 372 (285-480) | 1.67 | 154 (85-213) | 248 (146-379) |
|  | % (IQR) | 70% (64-75%) | 40% (34-44%) | 26% (20-29%) |  | 8% (7-14%) | 18% (13-23%) |
| Count (cell/µl) p-value | | 0.001 | 0.003 | 0.08 |  | 0.006 | 0.7 |
| Disease course/severity | | **Recovered** | | | | | |
|  |  | **CD3** | **CD4** | **CD8** | **CD4/CD8** | **B** | **NK** |
| Moderate | Median cell/µl (IQR) | 1353 (1052-1677) | 786 (593-1034) | 467 (350-696) | 1.69 | 186 (128-274) | 337 (204-487) |
|  | % (IQR) | 71% (65-76%) | 41% (35-47%) | 26% (20-32%) |  | 9.8% (8-13%) | 17.5% (12-24%) |
| Severe | Median cell/µl (IQR) | 1419 (1169-1746) | 757 (673-914) | 566 (391-808) | 1.60 | 223 (158-291) | 398 (198-559) |
|  | % (IQR) | 70% (48-74%) | 39% (29-45%) | 25% (22-34%) |  | 11% (8-14%) | 17.3% (13-24%) |
| Count (cell/µl) p-value | | 0.5 | 0.7 | 0.09 |  | 0.2 | 0.4 |
| Normal Range | | 850 - 2250 | 500 - 1450 | 160 - 950 | 1.0 - 3.0 | 100 - 500 | 60 - 450 |
|  |  | 62 - 81% | 32 - 59% | 15 - 36% |  | 8.0 - 20.0% | 4.0 - 22.0% |

IQR: Interquartile range
