# Supplementary material for: Longitudinal dynamics of SARS-CoV-2-specific cellular and humoral immunity after natural infection or BNT162b2 vaccination
Source: PLoS Pathog. 2021 Dec 28;17(12):e1010211. doi: 10.1371/journal.ppat.1010211 (PMC8757952; doi:10.1371/journal.ppat.1010211)
Supplement: Attachment — Submitted filename: Response to reviewers.pdf [file ppat.1010211.s008.pdf]

Editorial Comments

Thank you for submitting your manuscript. The reviewers appreciated your study and the need to validate findings in different cohorts. However, they had a major concern about the lack of novelty. If you are able to address this concern, perhaps by including additional data (such as the virologic parameters of infected patients, as suggested by Reviewer 1), or new immunologic results, we would be happy to reconsider this manuscript for publication.

Answer:

Thank you for the opportunity to send a revised version of the manuscript. We have answered to all the comments made by the reviewers, including additional data on viral load of infected patients and a description of the novelty of our work.

Reviewer's Responses to Questions

Part I – Summary

Please use this section to discuss strengths/weaknesses of study, novelty/significance, general execution and scholarship.

Reviewer #1: This manuscript describes the kinetic of the development of humoral and cellular immune response in a cohort of 88 patients with mild, moderate and severe COVID-19 immediately after symptoms onset until recovery or fatal outcome. They also analyzed the SARS-CoV-2 specific immune response in a large group of convalescents and in some vaccinated individuals.

The work provides evidences that a development of a coordinated functional T and B cell response is associated with mild disease. Even though these findings are not novel (see ref 15 and 16), I agree with the authors that previous work was done in limited number of patients and as such this work constitutes an important confirmation of such data.

Answer:

As mentioned by the reviewer, this work provides robust evidence that the early development of a coordinated functional T and B cell response upon SARS-CoV-2 infection is associated with mild disease.

We recruited 88 patients with acute infection at their arrival to the emergency room, after confirmation of SARS-CoV-2 infection. The peak disease severity of these patients was mild in 32, moderate in 34 and severe in 22. This patient cohort is much more comprehensive than the already published works by Tan *et al.* (ref 16 in the original paper: 12 patients of which 8 mild and 4 moderate/severe), Rydyznski Moderbacher *et al.* (ref 15 in the original paper: 24 patients of which 2 mild, 11 moderate and 11 severe, according to our severity classification) and Zhou *et al.* (ref 17 in the original paper: 17 patients of which 11 mild and 6 severe). The larger size of our cohort, the fact that patients were recruited immediately after symptom onset before any immunomodulatory treatment was administered and the balanced diversity of disease severity, allowed us to establish the prognostic value of the specific T cell response measured at the emergency room. In addition, this comprehensive cohort allowed us to perform a

multivariate analysis which determined that the specific T cell response measured at the emergency room is a protective factor irrespective of the patient's age and sex.

Thus, our work not only confirms the protective role of a prompt SARS-CoV-2-specific cellular immune response induction in naturally infected patients indicated by previous studies performed with a limited number of patients, but it goes beyond and establishes the prognostic value of the specific T cell response measured at the emergency room.

Please, read also other novel aspects of our work in response to Question 1 from Reviewer 2.

Reviewer #2: Almendro-Vázquez and colleagues investigated longitudinal kinetics of SARS-CoV-2-specific humoral and cellular immunity after either natural infection or BNT162b2 vaccination.

The authors analyzed S1-, M- and N-specific IFN-γ and IL-2 T cell immune responses and anti-S total and neutralizing antibodies in mild, moderate or severe acute COVID-19 patients.

They compared immune responses in COVID-19 patients with mild and severe disease. They also found a robust Th1-driven immune response in uninfected blood donors following BNT162b2-vacination. While understanding immune responses in COVID-19 is of a great importance, the novelty aspects of this study are unclear.

Specific comments:

1. The authors should specify upfront the novel aspect of their study in context of the literature.

Answer:

We very much appreciate the constructive comments of the reviewer. In the reviewed version of our manuscript, we have identified the novelties of our work and presented them together, in a summary form, in an earlier section of the manuscript (last paragraph of the Introduction section). Thus, the former last paragraph of the Introduction (lines 116-132):

"Here we longitudinally characterized the adaptive immune responses during acute and convalescent COVID-19 phases in three well-defined patient cohorts of different severity, in recovered patients up to 7 months PSO and in uninfected subjects up to three months after full vaccination. Our results confirm the protective role of a prompt SARS-CoV-2-specific cellular immune response induction in naturally infected patients and show the simultaneous development of a robust antigen-specific cellular and IgG response after BNT162b2 vaccination, which persists at least three months after vaccination"

Has been changed for:

"Here we longitudinally characterize the adaptive immune responses during acute and convalescent COVID-19 phases in three well-defined patient cohorts of different severity, in recovered patients up to 7 months PSO and in uninfected subjects up to three months

after full vaccination. Our acute infection cohort represents the largest of its kind, with 88 patients recruited before any medication was administered (in particular no immunomodulatory drugs) and has allowed us to statistically demonstrate that the presence of specific T cells against SARS-CoV-2 when patients arrive to the emergency room is a protective factor against developing severe COVID-19, independently of the age and gender of the patient. We also describe a correlation between higher numbers of specific T cells and lower viral load at diagnosis, which could explain the protection conferred by T cells. In addition, we characterize the vaccine-elicited adaptive immunity at five time-points and show robust cellular and humoral immune responses persist 3 months after real-world vaccination."

The present work is a thoroughly designed study to provide a comprehensive understanding of both arms of the adaptive immunity after SARS-CoV-2 infection or vaccination. We would like to describe in detail hereunder the several ways in which our work has added value compared to previous literature:

- The size of our cohort is the largest (N=88) and it has a balanced distribution according to disease severity (32 mild patients, 34 moderate and 22 severe). The work by Tan *et al.* had 12 patients of which 8 were mild and 4 moderate/severe, Rydyznski Moderbacher *et al.* had 24 patients of which 2 were mild, 11 moderate and 11 severe, and Zhou *et al.* had 17 patients of which 11 were mild and 6 severe. This extensive cohort has allowed us to provide a robust confirmation of previous results as well as further characterize the immune response to SARS-CoV-2.

- To our knowledge, this is the first study to statistically demonstrate that the presence of specific T cells against SARS-CoV-2 when patients arrive to the emergency room is a protective factor against developing severe COVID-19, independently of the age and gender of the patient, which are two major known contributors to disease outcome. Due to the larger nature of our cohort, and the variety of disease severity, we have been able to apply a multivariate analysis and establish the prognostic value of the specific cellular response.

- We made an arduous effort to recruit an early patient cohort, as close as possible to the start of their symptoms in order to be able to define the adaptive immune response from the beginning of its development. In our cohort, recruitment and first blood sample collection occurred between 4 and 11 (median 7) days post-symptom onset (DPSO). This is clearly sooner than in the work by Rydyznski Moderbacher *et al.* (blood collection 4-37 DPSO, median 11), and Zhou *et al.* (1-42 DPSO, median 14), and similar to Tan *et al.* (3-14 DPSO, information extracted from graphs).

- This early recruitment made possible to draw the first blood sample before any medication was administered at the hospital, importantly before any immunomodulatory treatment was given. Tan *et al.* specified that none of their patients received immunomodulatory treatments during the study period, however, in the other two studies this information is not provided and given that blood was collected from patients up to 37 and 42 DPSO, it is likely that some patients may have received immunomodulatory treatments prior to blood test. The absence of any interaction with immunomodulatory drugs prior to the blood collection at the emergency room in our cohort, has allowed the results to more clearly reflect the natural history of the immune response in COVID-19 and to reinforce the importance of this response in determining the course and outcome of the infection.

- Several studies have shown a strong humoral and cellular response in convalescent patients (refs 9-14), however, this occurred after many weeks of exposure to the virus. The early recruitment of our cohort has allowed us to define the immune response (or lack of) in 22 severe patients, of whom 8 did not survive the infection. Severe patients had a remarkably low cellular immune response against all 3 proteins, within the first 14 DPSO, which did not correlate with DPSO. Unlike mild and moderate patients, most severe patients lack a positive cellular response at the emergency room arrival (Figure 4A and 5H). The development of the cellular response was very slow, as it had still not increased after a week of hospitalization (Figure 4C). Upon arrival to ER, severe patients showed a negative correlation between antibody levels and some compartments of the cellular response, which reflected a discoordination between both arms of the immune response and the dominance of the humoral over the cellular immunity (Figure 4B).

- This is, together with the study by Tan *et al* (N=12), the only longitudinal study of the immune response from disease onset. Most our patients had one or two follow-up samples taken. Hospitalized patients (moderate and severe) had a second sample taken one week after hospitalization. Mild and moderate patients had another sample taken after recovery, approximately 4 weeks after diagnosis. We have now emphasized the longitudinal nature of the study by showing representative examples of the dynamics of cellular and humoral responses in mild, moderate and severe patients (Figure 5G).

- As another novelty in the manuscript, we have now added virological data of our acute infection cohort. We have found a correlation between the specific T cell response and the Ct value from the diagnostic RT-PCR, suggesting that patients with stronger specific T cell responses are more able to control viral replication (New Figure 5C and 5D).

- To our knowledge, this is the only study thoroughly comparing the magnitude and dynamics of the cellular and humoral immune responses after vaccination to those developed after natural infection. After the submission of our paper, an interesting work has been published by Tan et al (https://doi.org/10.1172/JCI152379, ref 45 in the revised manuscript) focusing on the cellular immune response in COVID-19. They describe "a similar mean magnitude of T cell responses between the vaccinees and SARS-CoV-2 convalescents 3 months after vaccination or virus priming" which coincides with our result in lines 381-383: "After the peak, the cellular response started to decline and 3 months after vaccination it was similar to that of COVID-19 recovered patients 4-7 months after infection". However, our manuscript also includes a direct comparison of cellular and humoral responses between infected patients (acute, convalescent and recovered) and vaccinees (peak and 3 months post-boost responses) (Figures 7M, 7N, 7O).

- Regarding the vaccine immunogenicity data, we consider the longitudinal data (especially regarding the cellular response) up to 3 months post-boost presented in this manuscript is of relevance. It is of great importance to acquire knowledge on the maintenance of specific immunity against SARS-CoV-2 following vaccination to help guide vaccination strategies. Given our results and those from Tan et al (ref 45), it is also clear that both humoral and cellular responses should be analysed and taken into account. We have performed a PubMed search under the terms "BNT162b2 AND immunity" up to November 3rd and found 881 results. There is growing literature about the immune response elicited by the vaccine measured 2 to 3 weeks post-boost. However, publications on the maintenance of this response are much scarcer. The published data from clinical trials cover the maintenance of humoral and cellular responses up to two months post-boost (Sahin et al, doi.org/10.1038/s41586-021-03653-6).

Real-world vaccination data on antibody maintenance is becoming more abundant, with several publications showing decline but in general persistence of specific antibodies 6 months after vaccination. This information has been updated in the Introduction of the revised manuscript, where the following sentence (lines 111-115): *"Nevertheless, there are scarce studies on the immunogenicity of BNT162b2 which expand up to a month post-vaccination [25-27] and data on the dynamics and long-term duration of SARS-CoV-2 specific T cells, antibodies and their neutralizing capacity after real-world vaccination are still lacking."*

Has been replaced for: *"There are studies on the immunogenicity of BNT162b2 which expand up to a month post-vaccination [25-27] and on the maintenance of vaccine-induced humoral responses six months post-vaccination [28,29], however, data on the dynamics and long-term duration of SARS-CoV-2 specific T cells after real-world vaccination are still lacking."*

However, there are only 3 publications with cellular response data beyond one month after real-world vaccination (Tan et al: doi.org/10.1172/JCI152379; Tober-Lau: doi.org /10.1016/S2213-2600(21)00456-2; Rossi et al: doi.org/10.3390/vaccines9101164), none of which study the dynamics of the vaccine-elicited cellular response in a longitudinal cohort. In contrast to the currently published literature, we recruited a longitudinal cohort of SARS-CoV-2 naïve individuals and measured their specific T cells at 5 timepoints, from pre-vaccine to 3 months post-boost, which allowed a more comprehensive characterization of the development and maintenance of the specific T cell response.

- Finally, some of the added value of this work to the published literature relies on our thorough methods to study the cellular and humoral immune response. Regarding the analysis of the cellular immune response, we have used the FluoroSpot method to analyse IFN-γ and IL-2-producing SARS-CoV-2-specific T cells, and we have been able to enumerate bifunctional IFN-γ+IL-2 SARS-CoV-2-specific T cells. Few papers have analysed the secretion of cytokines other than IFN-γ by SARS-CoV-2-specific T cells.

Apart from determining the frequency of antigen-specific T cell clones (SFU), we have obtained a parameter called relative spot volume (RSV) which represents the amount of secreted analyte (either IFN-γ or IL-2). This three-dimensional analysis of the volume of each spot provides further detail in the characterization of T cell responses. For example, we have found that in addition to developing specific T cell clones faster and in higher numbers, specific T cells from milder patients were more functional than those from severe patients, as shown by a higher amount (higher RSV) of IFN-γ and IL-2 secreted per clone (this information has now been added in Fig. 5B and Fig. S3I-K). To our knowledge, this is the first study to provide data on the relative spot volume of SARS-CoV-2-specific T cell responses.

Regarding the analysis of the humoral immune response, we have studied the production of SARS-CoV-2 specific antibodies not only by ELISA as most previous works, but also by using a newly developed high-sensitivity flow cytometry method (Hondler et al. See lines 525-532 in Methods section) which is more sensitive and correlates better with the neutralization capacity of antibodies than conventional ELISA. In addition, we have assessed the neutralization capacity of antibodies developed after infection and after vaccination using a classical neutralization assay with pseudotyped virus, which is a more established method than the surrogate virus neutralization assays used in other SARS-CoV-2 publications. Altogether, this shows that we have performed a rigorous and in-depth analysis of both arms of the adaptive immune response to SARS-CoV-2 infection and vaccination.

2. Abstract: “Description of the immune response elicited by real-world anti-SARS-CoV-2 vaccination is still lacking”: there are numerous publications on immune responses following COVID-19 vaccine. This sentence should be rephrased.

Answer:

As suggested by the reviewer, the following sentence (lines 54-56):

“Description of the immune response elicited by real-world anti-SARS-CoV-2 vaccination is still lacking.”

Has been changed for:

“Description of the long-term maintenance of both cellular and humoral responses elicited by real-world anti-SARS-CoV-2 vaccination is still scarce.”

3. Statistics on the graphs: there is no need to show 'ns' for not significant differences as these subtract from clear visualisation of significant differences.

Answer:

A suggested by the reviewer, we have eliminated most of the ‘ns’ shown in the graphs to allow better visualisation of significant differences. We have eliminated them from Figure 4A and 4D, Figure 5A, 5E and 5F, and Figure 7M. A few ‘ns’ have been left where we understood it was necessary to show the lack of statistically significant differences.

4. Representative T cell Fluorospots appear to be too dark thus lack clarity.

Answer:

We agree with the reviewer that the representative images of FluoroSpot appeared too dark and could not be fully appreciated in the way they were initially submitted. We still think they are informative examples and have now put them all together in larger size as Supplementary figure 2 (see below). We hope that the current quality of the pictures allows to visualize the difference between mild, moderate and severe patients.

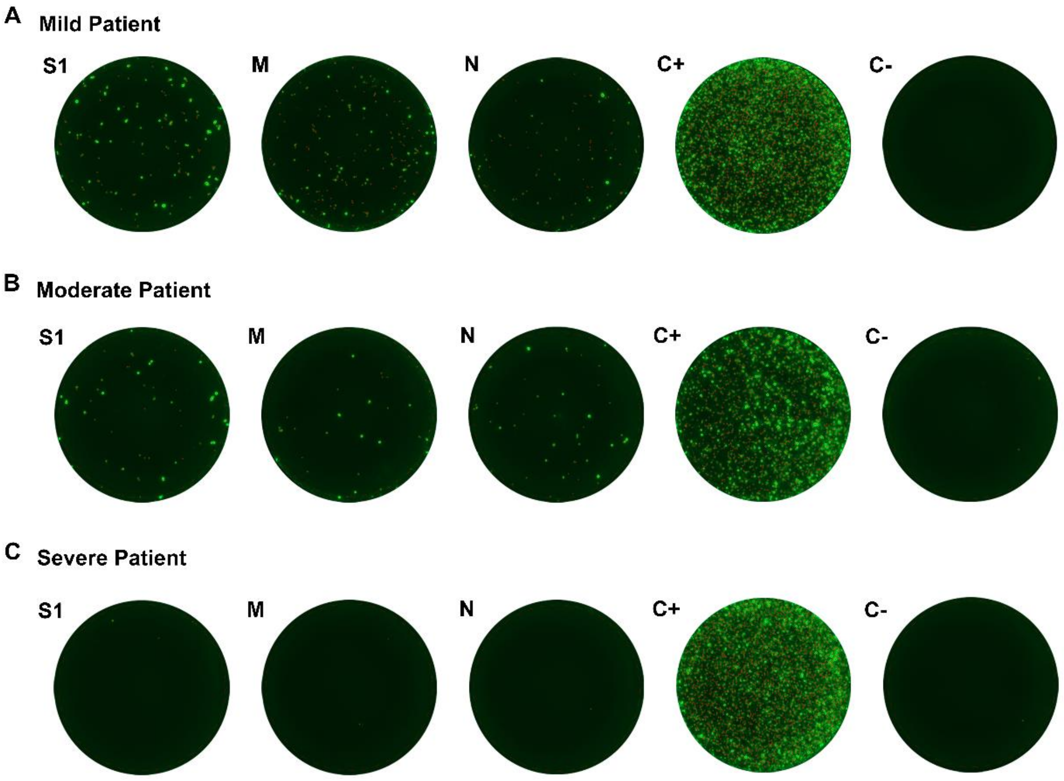

In order to respond to this comment, and to comments made by Reviewer 1, we have removed and/or added panels to some figures. We have therefore modified the figure legends and the references to the figures throughout the manuscript accordingly.

Part II – Major Issues: Key Experiments Required for Acceptance

Please use this section to detail the key new experiments or modifications of existing experiments that should be <u>absolutely</u> required to validate study conclusions.

Generally, there should be no more than 3 such required experiments or major modifications for a "Major Revision" recommendation. If more than 3 experiments are necessary to validate the study conclusions, then you are encouraged to recommend "Reject".

Reviewer #1: I have very little to say about the methods. Overall the data support the conclusions made.

A) However a weakness of the work is the lack of any virological quantification. It would be nice to add some virological parameters present in the studied patients. Quantity of virus present at the onset or a longitudinal analysis the persistence of SARS-CoV-2 +PCR over time in the different cohorts or in selected individuals might be a nice addition.

Answer:

As suggested by the reviewer, we have added some virological quantification from the acute SARS-CoV-2 infection cohort at the time of diagnosis. In this study, SARS-CoV-2 infection was confirmed in the 88 acute patients by either real time RT-PCR (N=44) or antigen testing (N=44). In the four months that acute patients were recruited, different real time RT-PCR assays were used. To make results comparable, we have now retested by the same real time RT-PCR assay all the samples that we had stored and have obtained their Ct values, which we have used as a relative measure of viral load. Unfortunately, during the hectic situation of the second pandemic wave in Spain not all samples were stored after diagnosis and we have not been able to calculate a Ct value for a large proportion of our patients. We have retested the nasopharyngeal samples obtained from 18 patients at the emergency room: 2 mild, 9 moderate and 7 severe.

These new virological data have been added in the Results section (lines 246-255) as follows:

"In our cohort, we did not observe an association between viral load at diagnosis and disease severity (Fig. 5C). Nevertheless, we found a strong correlation, mostly in moderate patients (r=0,98, p<0.0001), between a more potent specific T cell response and a higher Ct value at diagnosis, suggesting that patients with stronger specific T cell responses are more able to control viral replication (Fig. 5D)."

The data are shown in the new New Figure 5C and 5D:

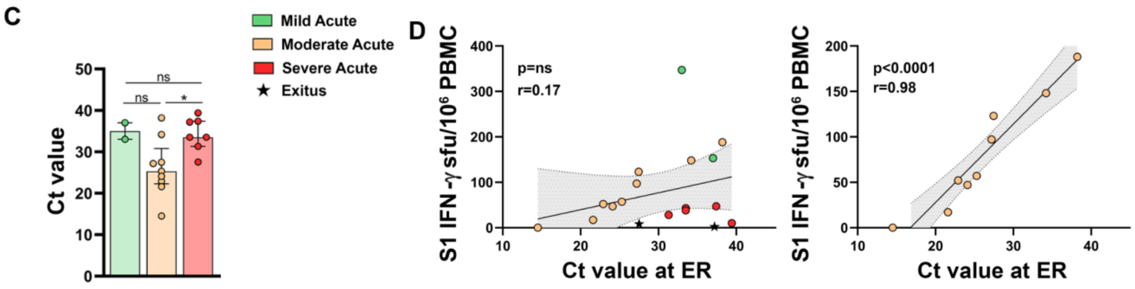

And described accordingly in their figure legend (lines 819-822):

*"(**C**) Ct values obtained on real time RT-PCR for detection of the E gene upon arrival to ER in mild, moderate and severe patients. A higher Ct value corresponded to a lower viral load. (**D**) Correlation between S1 IFN-γ-producing T-cells and the relative viral load represented as Ct value at ER."*

Finally, the Methods section (lines 550-557) has been updated accordingly:

Viral load quantification

"SARS-CoV-2 viral load at diagnosis was assessed using cycle threshold (Ct) values from a RT-PCR assay applied to nasopharyngeal swab samples. The RT-PCR assay was a laboratory-developed test (LDT) based on real time RT-PCR for detection of the E gene on the Panther Fusion Hologic (San Diego, CA, USA) using its open access functionality as previously described [48,49]. Amplification Ct values were considered a relative measure of viral load quantification. Lower Ct values corresponded with higher viral loads."

Due to the addition of this new virological data, we have added two new authors to the manuscript, Carmen Martín-Higuera and María Ángeles Meléndez-Carmona, from the Clinical Microbiology Department of our hospital.

B) Figures quality is very poor. The impression is that the authors displayed all the data that they have but this doesn't increase the clarity of the message. The display of the results of Elispot are of very poor quality and they don't deliver any message, they are black dots identical in all the figures. It will be also nice to show some longitudinal data of selected patients in the different cohorts and make a direct comparison between mild, moderate and severe. The data are shown now as a sort of cross-sectional analysis and it is not clear whether some patients were analyzed sequentially. It will be more indicative and clear to show some of individual representative patients. Furthermore, there are 8 fatalities in their acute severe cohort according to their table, but they did not highlight them in their graphs. I think they should be highlighted, as it will be interesting to know if their immunological response is any difference from the other severe patients

Answer:

Graphs have been created using GraphPad Prism version 8.0 software (GraphPad Software Inc, LaJolla, CA) and R software v4.0.3. Then, figures have been mounted in Adobe Photoshop CC 2015 and saved in high quality. Nonetheless, we will work with the editorial team to ensure a high quality in the final figures.

We agree with the reviewer that the representative images of FluoroSpot could not be fully appreciated in the way they were initially submitted. We still think they are informative examples and have now put them all together in larger size as Supplementary figure 2 (see below). We hope that the current quality of the pictures allows to visualize the difference between mild, moderate and severe patients.

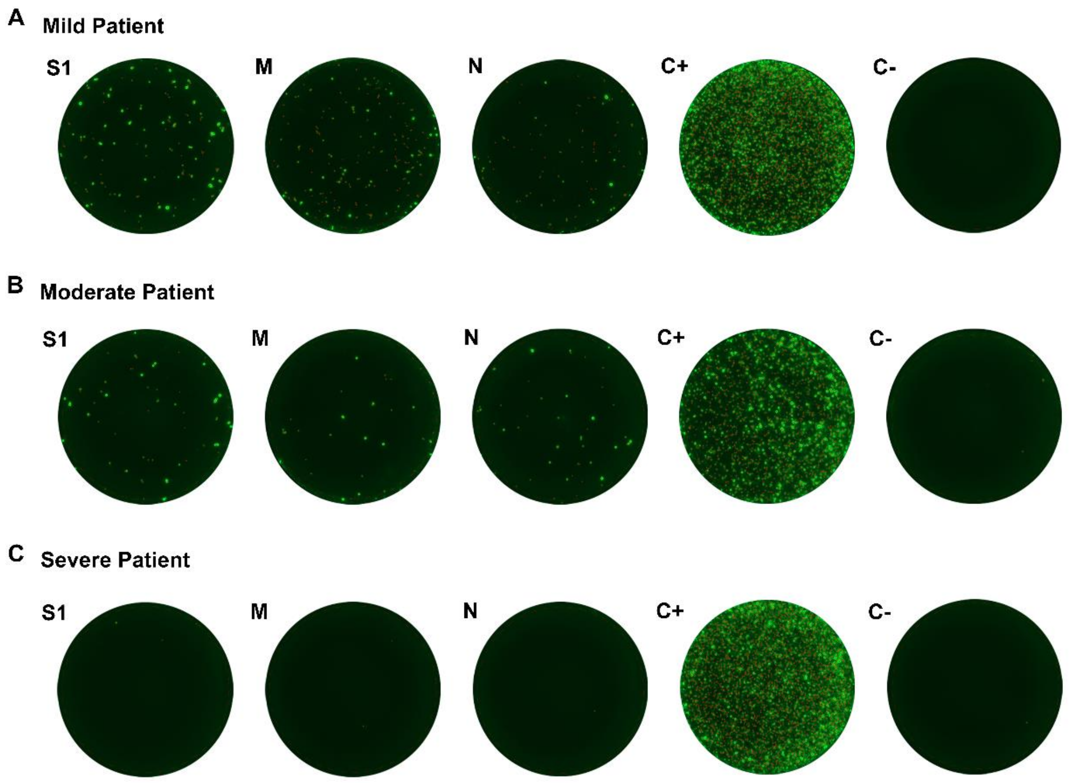

In order to respond to this and other comments, we have removed and/or added panels to some figures. We have therefore modified the figure legends and the references to the figures throughout the manuscript accordingly.

Regarding the longitudinal nature of the study, naturally infected patients and vaccinees are followed up overtime. This is shown in Figure 1: at least two samples were obtained from patients belonging to the acute infection cohort and five sequential samples were obtained from vaccinated individuals. The sampling schedule is also described in the first Results section:

Lines 145-147: *"In moderate and severe patients, a follow-up sample was taken a week after hospitalization. In mild and moderate patients another sample was taken during the convalescent phase, a month PSO."*

Lines 161-164: *"Lastly, vaccinated individuals were 28 healthcare workers, without previous SARS-CoV-2 infection (Fig. 1C). They received two 30 µg doses of BNT162b2 (Pfizer-BioNTech), 21 days apart. Samples were obtained pre-vaccine, pre-boost and 15, 30 and 90 days after completing vaccination."*

To emphasize the longitudinal nature of the study, we have added the term "sequentially" in the beginning of the description of the development of the specific immune response during acute infection, in Results section, lines 170-172:

*“SARS-CoV-2-specific T cells, IgG and neutralizing antibodies were **sequentially** analyzed in patients with natural infection, at their arrival to ER and during convalescence.”*

Moreover, as suggested by the reviewer, we have added representations of longitudinal cellular and humoral data from individual representative patients to allow a direct visual comparison of the dynamics of adaptive immunity between mild, moderate and severe COVID-19 patients. These representations have been added as Figure 5G:

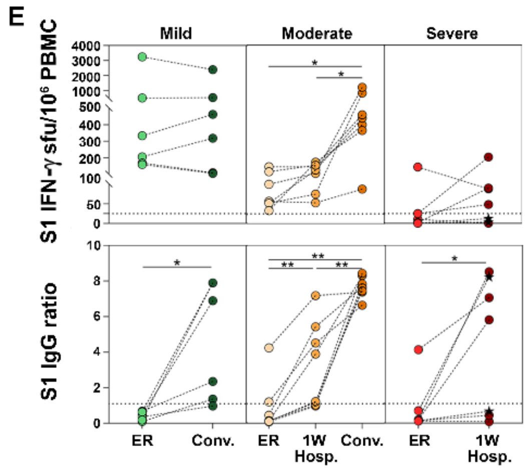

And have been mentioned it in the Results section (lines 257-259) as follows:

“The different dynamics of cellular and humoral immune response development are shown with six representative patients from each of the 3 cohorts in Fig. 5G.”

Regarding the fatalities, we have now highlighted them by representing them as stars instead of dots in Figure 4. Interestingly, by highlighting the severe patients who died, it becomes obvious that these patients develop an even weaker and/or slower specific T cell response than severe patients who survived. We have included this new analysis in the manuscript (lines 225-227) as follows:

“Eight of the 22 (36%) severe patients died during hospitalization. These patients who died had significantly lower cellular response against all 3 proteins (Fig. 4H) and a trend towards lower humoral response (Fig. 4I) than severe patients who survived.”

We have also included the following two new panels in Figure 4 showing the comparison in cellular (Fig. 4H) and humoral (Fig. 4I) response between severe patients who survived and those who did not:

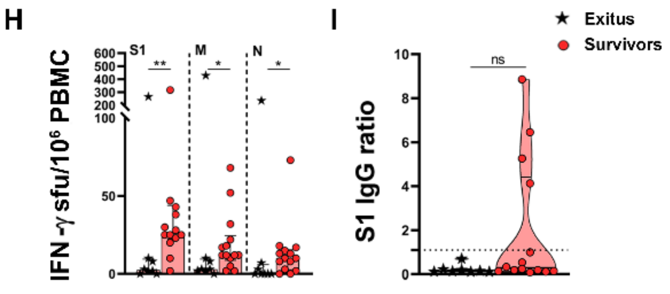

Part III – Minor Issues: Editorial and Data Presentation Modifications

Please use this section for editorial suggestions as well as relatively minor modifications of existing data that would enhance clarity.

Reviewer #1: The T cell response to Spike was analysed only using peptides covering S1 regions. This should be discuss as a potential limitation of the analysis.

Answer:

We agree with this comment made by the reviewer, and have added it as a limitation in the Discussion section (lines 466-468) as follows:

"It should also be noted that the T cell response was analysed using peptides covering the S1 region, which contains the receptor binding domain, but the potential response against S2 region was not analyzed."

The authors should acknowledge in the results and not only in the introduction, the fact that their data confirmed previous works, I don't think the authors can state that "This is the first study analyzing the SARS-CoV-2-specific cellular and humoral immune response in patients upon ER arrival". This was also done in ref 15-16

Answer:

As suggested with the reviewer, we have referenced the existence of previous works with similar results:

- In Results section (lines 254-255) the following sentence has been added: *"These results confirm and further validate smaller studies which had indicated that the development of a functional T cell response is associated with mild disease."*

- In Discussion section (lines 399-402) the sentence *"This is the first study analyzing the SARS-CoV-2-specific cellular and humoral immune response in patients upon ER arrival, which allowed the characterization of both arms of adaptive immunity from the start of natural infection without any interference from immunomodulatory medication."*

has been changed for *"To date, this is the largest study analyzing the SARS-CoV-2-specific cellular and humoral immune response in patients upon ER arrival, which allowed the characterization of both arms of adaptive immunity from the start of natural infection without any interference from immunomodulatory medication."*

- In Discussion section (lines 439-442) the following sentence has been added: "*This coincides with the recent report by Tan et al [45] in which a similar magnitude of T cell responses between the vaccinees and recovered patients was observed three months after vaccination or infection."*

Reviewer #2: (No Response)
