# Supplementary material for: Longitudinal dynamics of SARS-CoV-2-specific cellular and humoral immunity after natural infection or BNT162b2 vaccination
Source: PLoS Pathog. 2021 Dec 28;17(12):e1010211. doi: 10.1371/journal.ppat.1010211 (PMC8757952; doi:10.1371/journal.ppat.1010211)
Supplement: Attachment — Submitted filename: Responses to review comments.pdf [file ppat.1010211.s009.pdf]

Responses to comments on PPATHOGENS-D-21-01759R1:

“Longitudinal dynamics of SARS-CoV-2-specific adaptive immunity after natural infection or BNT162b2 vaccination”

and description of the changes made in the manuscript.

Comment from the Editor:

Thank you for responding to the reviewers’ comments by adding new data and highlighting how your study is different from published ones. However, the discussion of the literature could be improved. In particular, several groups have now examined the durability of vaccine-induced immune responses beyond 3 months.

Answer:

We appreciate this constructive suggestion that improves our manuscript. The following paragraph has been added in the Discussion section (lines 439-450), including five new references (references 46, 47, 48, 49 and 50):

“So far, several publications have investigated the durability of vaccine-induced humoral responses up to 6 months after vaccination. A gradual and substantial decrease in IgG specific antibodies and neutralizing antibody levels has been reported through the first half a year post-vaccination [28,46,47], independently of having received the mRNA based BNT162b2 or the adenoviral vector-based ChAdOx1 nCoV-19 [48]. Both, BNT162b2-induced SARS-CoV-2 antibody and T-cell responses have been shown to experience a more important decline after 6 months in the elderly than in young health-care workers [49]. Although these observations support the recommendation for booster vaccinations, the effect of antibody waning needs to be clearly determined, as, importantly, specific memory B cells remain 6 months after the immunization [29] or after the natural infection, even in the absence of detectable neutralizing antibodies [50].”
